# Pain in the Side

**Published:** 1847-04

**Authors:** 


*Pain in the Side*, in thoracic inflammations, generally corresponds, according to the indication of the patient, not to the precise point of the organ affected, but to one a little below it,—that is, the painful sensation experienced is in a situation inferior to the lesion. When local evacuations of blood, therefore, are ordered, or blisters, they should be directed to be applied a little higher than the painful part—(*Rostan.*) This precision is not without importance in certain cases, for it may happen that, following the indication of the patient, remedies are often applied to the abdomen, when the disease is at the lower part of the chest.—*Ibid.*

